# Comparison of analgesic effects of percutaneous and transthoracic intercostal nerve block in video-assisted thoracic surgery: a propensity score-matched study

**DOI:** 10.1186/s13019-024-02490-8

**Published:** 2024-01-30

**Authors:** Hongliang Hui, Haoran Miao, Fan Qiu, Huaming Li, Yangui Lin, Bo Jiang, Yiqian Zhang

**Affiliations:** Department of Thoracic Cardiovascular Surgery, The Eighth Affiliated Hospital of Sun Yat-sen University, No 3025 Shennan Middle Road, Shenzhen, 518033 Guangdong P.R. China

**Keywords:** Analgesia, Intercostal nerve block, Video-assisted thoracoscopic surgery

## Abstract

**Background:**

This study aimed to compare the analgesic efficacy of transthoracic intercostal nerve block (TINB) and percutaneous intercostal nerve block (PINB) for video-assisted thoracic surgery (VATS) using a retrospective analysis.

**Methods:**

A total of 336 patients who underwent VATS between January 2021 and June 2022 were reviewed retrospectively. Of the participants, 194 received TINB and were assigned to the T group, while 142 patients received PINB and were assigned to the P group. Both groups received 25 ml of ropivacaine via TINB or PINB at the end of the surgery. The study measured opioid consumption, pain scores, analgesic satisfaction, and safety. Propensity score matching (PSM) analysis was performed to minimize selection bias due to nonrandom assignment.

**Results:**

After propensity score matching, 86 patients from each group were selected for analysis. The P group had significantly lower cumulative opioid consumption than the T group (*p* < 0.01). The Visual Analogue Scale (VAS) scores were lower for the P group than the T group at 6 and 12 h post-surgery (*p* < 0.01); however, there was no significant difference in the scores between the two groups at 3, 24, and 48 h (*p* > 0.05). The analgesic satisfaction in the P group was higher than in the T group (*p* < 0.05). The incidence of back pain, nausea or vomiting, pruritus, dizziness, and skin numbness between the two groups was statistically insignificant (*p* > 0.05).

**Conclusion:**

The study suggests that PINB provides superior analgesia for patients undergoing thoracic surgery compared to TINB without any extra adverse effects.

**Supplementary Information:**

The online version contains supplementary material available at 10.1186/s13019-024-02490-8.

## Background

Over the last few decades, several efforts have been made to develop more effective analgesia approaches for postoperative pain management [1, 2]. Multimodal anesthesia based on regional nerve blocks is typically preferred over general anesthesia [3]. There are primarily four regional nerve block approaches that are commonly used: thoracic epidural anesthesia, thoracic paravertebral nerve block, erector spinae block, and intercostal nerve block [4–6]. Thoracic epidural anesthesia, which was previously considered the gold standard for postoperative analgesia after thoracoscopy [7], is associated with various complications, including dural puncture, nerve injury, respiratory depression, and paraplegia [8]. Paravertebral nerve block and erector spinae block, on the other hand, often require ultrasound guidance, and some patients may experience parasympathetic symptoms due to the puncture needle, such as hypotension, bradycardia, and even syncope [9, 10].

In recent years, intercostal nerve block has gained increasing attention for early postoperative analgesia after thoracic surgery due to its convenience, good analgesic effect, and limited complications [11, 12]. Therefore, intercostal nerve block is considered to be a reliable method for postoperative analgesia. However, there are several different intercostal nerve block approaches, including percutaneous intercostal nerve block (PINB) and transthoracic intercostal nerve block (TINB), and their effects on postoperative analgesia after minimally invasive thoracic surgery remain unclear. It has been observed that during TINB, a portion of the local anesthetic may leak along the ruptured pleura or injection site, and the absorption rate of the local anesthetic may vary depending on the injection location. Therefore, we hypothesized that the PINB approach may potentially offer a longer duration of postoperative analgesia than TINB. Thus, in this retrospective study, we aimed to analyze and compare the analgesic effects between PINB and TINB in video-assisted thoracoscopic surgery (VATS).

## Methods

This study adhered to the principles outlined in the Declaration of Helsinki (revised in 2013) and received approval from the ethics committee of the Eighth Affiliated Hospital of Sun Yat sen University (No. 2022-024-01), and the requirement for obtaining informed consent was waived.

### Study subjects

**Fig. 1:**
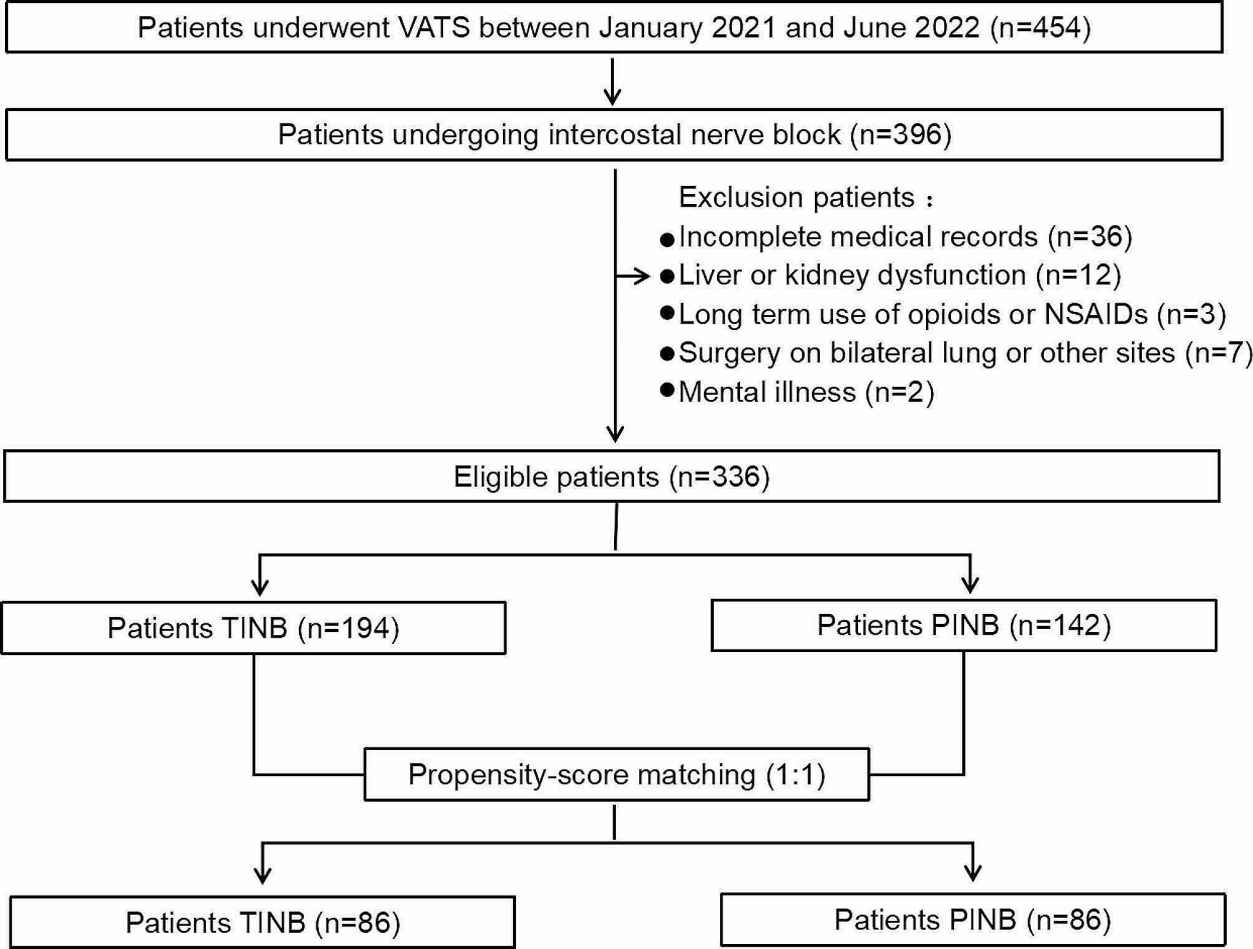
Flowchart illustrating the patient selection process. VATS video-assisted thoracic surgery, NSAIDs nonsteroidal anti-inflammatory drugs, TINB transthoracic intercostal nerve block, PINB percutaneous intercostal nerve block

**Fig. 2:**
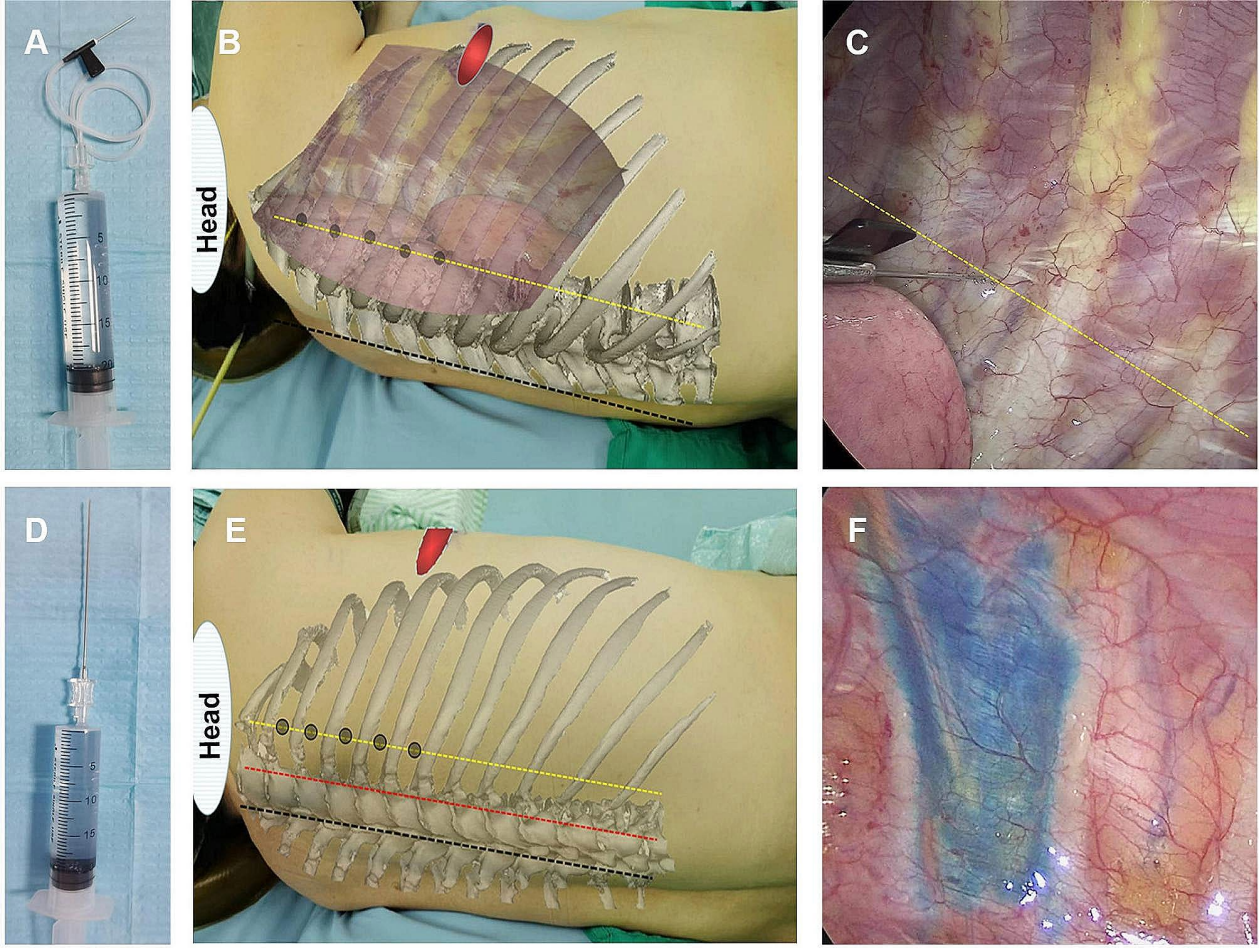
Transthoracic intercostal nerve block and percutaneous intercostal nerve block. **a** disposable intravenous infusion needle; **b** transthoracic intercostal nerve block sketch map, black line indicates posterior median line, yellow line indicates connecting line at 2 cm outside the sympathetic chain; **c** thoracoscopic view, yellow line indicates connecting line at 2 cm outside the sympathetic chain; **d** disposable anesthesia needle; **e** percutaneous intercostal nerve block sketch map, black line indicates posterior median line, red line indicates parallel line at 2.5 cm outside the posterior median line, yellow line indicates parallel line at 5 cm outside the posterior median line; **f** thoracoscopic view

We retrospectively collected clinical data from 454 consecutive patients who underwent thoracoscopic surgery between January 2021 and June 2022 (Fig. 1). Among them, 396 patients were randomly assigned to undergo percutaneous or transthoracic intercostal nerve block. The patients who received 25 ml of 0.5% ropivacaine (5 ml per intercostal space) injected under the pleura 2 cm outside the sympathetic chain via a disposable intravenous infusion needle (0.719TWLB, Shandong Weigao Group Medical Polymer Product Co., Ltd.) under thoracoscopy were assigned to Group T (Fig. 2A - C), while those who received 25 ml of 0.5% ropivacaine (5 ml per intercostal space) injected by percutaneous puncture 5 cm outside the midline of the spine using a disposable anesthesia needle (AN-S 0.90.9, Shanghai Aisil Medical Technology Co., Ltd.) were assigned to Group P (Fig. 2D - F). Ultimately, 336 patients were selected based on the following inclusion criteria: (1) patients who underwent uniportal thoracoscopic surgery following clinical diagnosis of lung malignancy or bullae, (2) patients aged between 18 and 75, (3) patients with complete medical records, including medical history, preoperative and intraoperative data, and (4) patients who underwent correct postoperative pain score assessments with complete documentation of opioid usage. The exclusion criteria for this study were as follows: (1) Patients with incomplete documentation of postoperative opioid dose and pain scores. (2) Patients with liver or kidney dysfunction affecting drug metabolism. (3) Patients with chronic pain history who have taken opioids or NSAIDs for an extended time. (4) Patients who had bilateral pulmonary surgery or surgery involving other parts of the body. (5) Patients with experience of mental illness that affect pain assessment.

### Anesthesia methods

After the patient entered the surgery room, we established venous access for the patient, and routinely monitored the vital indicators of electrocardiography (ECG), blood pressure (BP), pulse oxygen saturation (SpO_2_), respiratory rate (RR), and bispectral index (BIS). Anesthesia induction involved the intravenous administration of midazolam (0.04 mg/kg), propofol (1.0-1.5 mg/kg), fentanyl (0.4 µg/kg), and vecuronium (0.1 mg/kg). Anesthesia was maintained with propofol 3–6 mg/kg/h by bispectral index 40–60 and additional bolus doses of remifentanil 0.05–0.25 µg/kg/min to keep mean arterial pressure (MAP) or heart rate values around 20% below baseline values. All patients received patient-controlled intravenous analgesia (PCIA) after VATS. The PCIA protocol was programmed with fentanyl 1 mg diluted in 100 ml of 0.9% saline; 3 ml bolus, lockout time interval of 10 min and 1 h limit of 12 ml without any baseline infusion. Administer immediately if VAS ≥ 4 or as needed by the patient.

### Data collection

An Electronic Data Capture System was used by trained, designated investigational site personnel to collect and transfer study data from source records into common eCase Report Forms. Preoperative data of eligible patients, including gender, age, height, weight, reason for surgery were collected from the patient admission records. Intraoperative data obtained from anesthesia records and operation records included types of surgery and surgical time. Postoperative data, including cumulative opioid consumption, pain scores at different times, patient satisfaction with pain, and intercostal nerve block complications (back pain, nausea/vomiting, pruritus, bleeding/hematoma, dizziness, skin numbness, total morbidity) were collected from postoperative medical records and nursing records, course records, and pain evaluation forms.

The main outcome measure was postoperative opioid consumption, which was converted into morphine milligram equivalent and recorded during the initial 48 h postoperative. Dose conversion information was listed in Supplementary Table 1. The secondary outcome measures were the pain scores, analgesia satisfaction, and safety assessment. Postoperative pain was evaluated and scored in the two groups at 1, 3, 6, 12, 24, and 48 h postoperatively by VAS. Pain scales were numbered from 0 to 10, where score of 0 indicates no pain, whereas 10 indicates the worst pain. Analgesia satisfaction in the two groups was recorded 48 h postoperatively, with score of 0 indicating patient dissatisfaction while 1 indicating patient satisfaction.

### Statistical analysis

To minimalize the selection bias inherent in nonrandom assignment study, propensity score matching was used to remove basic demographic differences such as age, gender, BMI, reason for surgery, type of operation, and duration of surgery. Furthermore, logistic regression models were used to increase the between-patient similarity qualitatively. As a result, no additional confounder adjustment was needed when analyzing the matched sample. Patients with the closest propensity scores were paired using a 1:1 nearest neighbor matching algorithm. A caliper width of 0.02 units was used. The propensity score matching procedure yielded two matched cohorts of 86 patients in each. We then compared the standardized differences in P group and T group for all covariates between prematch and postmatch.

Continuous variables were expressed as mean values with a range of one standard deviation (SD) and were compared using an independent samples t-test. Continuous variables at different times were compared using two-way ANOVA followed by post hoc Bonferroni test. Categorical variables were represented numerically and were compared by Chi-square or Fisher’s exact test. All tests were 2-sided and were defined as significant at a *P* value less than 0.05. All calculations were performed using the SPSS statistical package (SPSS version 25.0; IBM Corp., Armonk, NY, USA).

## Results

### Patient enrollment

From January 2021 to June 2022, a total of 454 patients who underwent VATS were screened for eligibility in this study. Of those, 118 patients were excluded based on the defined criteria, resulting in a final sample size of 336 patients. Among these, 194 patients received TINB, and 142 received PINB. After propensity score matching, 86 patients were included in the analysis for each group (Fig. 1).

### Baseline data for patients in two groups

Comparison of patients’ clinical information between T and P groups before and after matching was shown in Table 1. Clearly, propensity score matching achieved a good balance in gender, age, BMI, smoking, drinking, hypertension, type of surgery, and the pleural adhesions between the two groups (*p* > 0.05). Hence, we carried out further analysis for the matched samples as described below.

**Table 1:** Baseline demographic and anesthetic data

Characteristics	Before PSM		*p*	After PSM		*p*
	T group *n* = 194	P group *n* = 142		T group *n* = 86	P group *n* = 86	
Age (years)	58.4 ± 14.8	43.9 ± 11.9	0.000*	46.8 ± 14.9	45.6 ± 13.2	0.578
Sex, n (%)			0.965			0.647
Male	102 (52.58)	75 (52.82)		41 (47.67)	44 (51.16)	
Female	92 (47.42)	67 (47.18)		45 (52.33)	42 (48.84)	
BMI	23.1 ± 2.8	23.6 ± 2.7	0.143	23.4 ± 2.6	23.3 ± 2.3	0.922
Reason for surgery, n (%)			0.114			0.934
Rupture of pulmonary bulla	19 (9.79)	32 (22.54)		15 (17.44)	13 (15.12)	
Giant pulmonary bulla	2 (1.03)	3 (2.11)		2 (2.33)	2 (2.33)	
Ground glass nodule	112 (57.73)	95 (66.90)		54 (62.79)	58 (67.44)	
Solid pulmonary nodule	61 (31.44)	47 (33.10)		15 (17.44)	13 (15.12)	
Types of operation, n (%)			0.000*			0.765
Bullae surgery	21 (10.82)	35 (24.65)		17 (19.77)	15 (17.44)	
Wedge resection	52 (26.80)	47 (33.10)		29 (33.72)	32 (37.21)	
Segmental resection	95 (48.97)	56 (39.44)		33 (38.37)	35 (40.70)	
Lobectomy	26 (13.40)	4 (2.82)		7 (8.14)	4 (4.65)	
Pleural rupture, n (%)	62 (31.96)	47 (33.10)	0.679	29 (33.72)	31 (36.05)	0.742
Duration of surgery (min)	79.4 ± 36.8	85.3 ± 29.9	0.118	80.6 ± 35.3	82.9 ± 27.4	0.640
Intraoperative opioid consumption, MME	87.3 ± 21.1	91.2 ± 23.2	0.107	87.2 ± 22.3	90.1 ± 23.0	0.426
Propofol, mg	427.0 ± 88.5	449.8 ± 74.7	0.014	435.0 ± 80.1	448.3 ± 65.8	0.255
Postoperative opioid consumption, MME	34.2 ± 7.5	32.9 ± 7.3	0.104	36.1 ± 5.6	30.8 ± 6.6	0.000*
Analgesia satisfaction, n (%)	129 (66.49)	106 (74.65)	0.107	52 (60.47)	69 (80.23)	0.005*

Values are expressed as means ± SD or numbers. ^*^p < 0.01

Abbreviations: VATS video-assisted thoracic surgery, PSM propensity score matching, BMI body mass index, MME morphine milligram equivalent

### Comparison of opioid consumption and satisfaction with analgesia

Opioid consumption, patient satisfaction, complications, and VAS were compared between the two groups at different time points after surgery. The postoperative opioid use within the first 48 h in P group was significantly less than in T group (*p* < 0.01). Consistently, postoperative pain patient satisfaction was higher in P group than in T group (*p* < 0.01) (Table 1).

### Comparison of postoperative VAS score

**Fig. 3:**
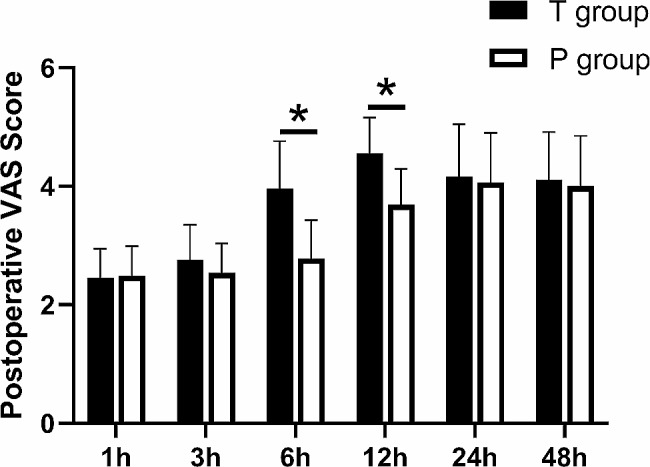
Visual analog scale scores at different times postoperative. At 6 and 12 h postoperatively, *p < 0.01 T group compared with P groups. VAS visual analog scale scores (0 ~ 10, 0 = no pain, 10 = most severe pain)

Two-factor repeated measures ANOVA (one interindividual factor + one intraindividual factor) was performed to compare the effect of a certain drug on reaction time (Table 2). We found patients with PINB had lower VAS scores compare to patients with TINB at 6 and 12 h after surgery (*p* < 0.01 and *p* < 0.01, respectively); however, there was no significant difference in VAS scores between the two groups at 1, 3, 24, and 48 h after surgery (*p* > 0.05) (Fig. 3).

**Table 2:** Analysis of variance of repeated measurement of pain level

Groups	1 h	3 h	6 h	12 h	24 h	48 h	F^1^	F^2^	F^3^
T group	2.45 ± 0.50	2.75 ± 0.60	3.96 ± 0.80	4.55 ± 0.61	4.16 ± 0.89	4.11 ± 0.81	502.70	194.59	49.67
P group	2.49 ± 0.50	2.54 ± 0.50	2.78 ± 0.65	3.69 ± 0.60	4.06 ± 0.84	4.00 ± 0.85			
t	-1.122	3.603	12.995	14.597	2.745	1.248			
*p*	0.637	0.017	0.000*	0.000*	0.468	0.395	0.000*	0.000*	0.000*

Values are expressed as means ± SD. F^1^, time effect F; F^2^, group effect F; F^3^, interaction effect F. ^*^p < 0.01

### Comparison of complications

There was no significant difference between P and T groups in complications including back pain, nausea or vomiting, pruritus, dizziness, skin numbness, and total incidence rate (*p* > 0.05), and the risk of hemorrhage or hematoma was lower in P group than in T group (*p* < 0.05) (Table 3).

**Table 3:** Complications of intercostal nerve block

Variable	T group	P group	*p*
	*n* = 86	*n* = 86	
Back Pain	2 (2.33)	1 (1.16)	1.000
Nausea/Vomiting	3 (3.49)	4 (4.65)	1.000
Pruritus	0 (0.00)	1 (1.16)	1.000
Hemorrhage/hematoma	17 (13.95)	6 (6.98)	0.014*
Dizziness	5 (5.81)	4 (4.65)	1.000
Skin numbness	2 (2.33)	3 (3.49)	1.000
Total incidence rate	24 (27.91)	19 (22.09)	0.379

Values are expressed as number and percentage. ^*^p < 0.05

## Discussion

Compared with thoracotomy, VATS has the advantages of smaller incision, less pain, and faster postoperative recovery [13, 14]. However, postoperative pain, especially intercostal neuralgia, is the main factor attributing to the poor patient experience, which needs to be addressed [2]. If intercostal neuralgia is not relieved in time after surgery, chronic pain will occur, which will significantly reduce patient’s quality of life [15, 16]. Therefore, effective analgesic measures are crucial to patients after video-assisted thoracoscopy. In the present investigation, a retrospective analysis was performed on the clinical data of patients who received TINB or PINB. It was identified that PINB yielded superior postoperative analgesic outcomes.

It is widely acknowledged that postoperative pain resulting from surgical trauma is influenced by various factors, including the patient’s pain sensitivity, psychosocial factors, anesthesia factors, and surgeon factors [2]. Several studies have attempted to alleviate postoperative pain from different perspectives, such as improving analgesic drugs, psychological interventions, prophylactic medication, enhancing analgesic methods, and utilizing adjunctive agents in anesthesia [17, 18]. This study aims to investigate the postoperative analgesic method of intercostal nerve block following single-port thoracoscopy from the perspective of surgical practitioners, under the premise of relatively invariant influences from other factors, in an attempt to identify a safer and more effective postoperative analgesic method.

Different postoperative analgesia strategies have been tested by thoracic surgeons and anesthesiologists [19], among which regional nerve block is increasingly used due to its superior convenience to implement intraoperatively [20]. There have been many comparative studies on intercostal nerve block, serratus anterior plane block, paravertebral nerve block, and epidural nerve block [21, 22]. The approaches for intercostal nerve block are mainly through percutaneously and transthoracicly. However, there are no comparative studies on the analgesic efficacy of the two routes of intercostal nerve block. Our study found that PINB provided better analgesia than TINB. There is a possible explanation to this difference. At the end of the surgery, PINB and TINB were performed by surgeon under thoracoscopic direct vision, both of which could accurately block the target intercostal nerve. However, part of the local anesthetic might flow along the ruptured pleura or injection hole, as a result, the exact dose in TINB could not be accurately determined. If the patient resumed spontaneous breathing after waking up, the intercostal muscles might be further squeezed, thereby leading to further loss of local anesthetic. In patients with bullae-pleurodesis or pleural adhesions, the integrity of the pleura is disrupted, and leakage is more pronounced with transthoracic intercostal nerve blocks. Previous studies have shown that the penetration of local anesthetic into the pleural space can cause irregular absorption of the drug into the bloodstream [23, 24]. In severe cases, patients may experience local anesthetic poisoning. Although there are many studies related to intercostal nerve block [11], there is no consensus on the choice of the local anesthetic [25].

While some studies have blocked 6 or more intercostal spaces (the wide block method) for adequate intercostal nerve block in no-single thoracoscopic surgery [26, 27], for single portal thoracoscopic surgery, we adopted the fourth or fifth intercostal incision and then used the surgical incision and the adjacent upper two and lower two intercostal space for intercostal nerve block. Our results showed that there was no significant difference in the analgesic effect between our method and the wide block method.Previous Studies have shown that effective intercostal nerve blocks can be maintained for 14 h after surgery [28, 29], which is consistent with our observations. In this study, the VAS of the patients in T group began to increase at 3 h and reached the highest level at 12 h post-surgery. Although the patients in P group also showed the same pattern, there was a notable difference between T and P groups. The VAS of the patients in P group went up more slowly than T group from 3 h post-surgery, exhibiting a significant difference from T group at 6 and 12 h after surgery. Yet, there was no significant difference between P and T groups at 48 h, suggesting that PINB has an advantage over TINB within the initial 12 h after surgery. Since previous studies have shown that adding adjuvants such as dexamethasone or dexmedetomidine to local anesthesia may extend the effective time of analgesia [30, 31], we will verify this effect in our further study.

Moreover, this study also compared the safety of PINB and TINB from the aspects of back pain, nausea or vomiting, pruritus, hemorrhage or hematoma, dizziness, delirium, and skin numbness. The results showed that PINB had obvious advantages over TINB in ​​hemorrhage or hematoma, while other differences were not statistically significant, indicating PINB as a relatively safe and reliable method of local anesthesia.

### Limitations

There were some limitations in this study. The major limitation was its retrospective design. Nonetheless, we performed propensity score matching to compensate and increase confidence. The results from this study would serve as the basis for further prospective randomized studies. In addition, in this study, we observed that patients’ pain scores were significantly different at 6 and 12 h after surgery, but we did not count opioid consumption at 6 or 12 h. It is conceivable that when patients feel pain, they will use more opioids to relieve the pain. As a result, increased opioid consumption will conceal the difference in the postoperative VAS score between the two groups, which should be assessed carefully. Lastly, due to the relatively small number of patients included in this study, further controlled study using large sample size will yield more convincing conclusions.

## Conclusions

In summary, this retrospective study suggests that PINB is more superior than TINB to provide effective pain control after minimally invasive thoracic surgery without additional adverse effects.

### Electronic supplementary material

Below is the link to the electronic supplementary material.

Supplementary Material 1

## Data Availability

The datasets used and/or analyzed during the current study are available from the corresponding author on reasonable request.

## Abbreviations

TINB: Transthoracic intercostal nerve block
PINB: Percutaneous intercostal nerve block
VATS: Video-assisted thoracic surgery
PSM: Propensity score matching
BMI: Body mass index
MME: Morphine milligram equivalent
NSAIDs: Nonsteroidal anti-inflammatory drugs
SD: Standard deviation
