# Supplementary material for: Comparison of analgesic effects of percutaneous and transthoracic intercostal nerve block in video-assisted thoracic surgery: a propensity score-matched study
Source: J Cardiothorac Surg. 2024 Jan 30;19:33. doi: 10.1186/s13019-024-02490-8 (PMC10829370; doi:10.1186/s13019-024-02490-8)
Supplement: Supplementary file 1 — Supplementary Material 1 [file 13019_2024_2490_MOESM1_ESM.docx]

| **Supplementary Table 1.** Conversion Factors for Morphine Milligram Equivalents | | |
| --- | --- | --- |
|  | **Route** | **Conversion (multiplication) factor** |
| Morphine | IV, IM, SC | 1 |
| Morphine | PO | 0.33 |
| Fentanyl | IV, PO, IM | 100 |
| Remifentanil | IV | 134 |
| IM, intramuscular; IV, intravenous; PO, oral; SC, subcutaneou | | |
